# Community perspectives on access to healthcare for the Rama indigenous people, Nicaragua: the utility of Cultural Safety outside high-income countries

**DOI:** 10.1186/s12939-026-02920-0

**Published:** 2026-06-25

**Authors:** Andrew Papworth, Guerry Windell

**Affiliations:** 1 School for Business and Society, University of York, York, UK; 2 Rama Community Leader, GTR-K, Bluefields, Nicaragua

**Keywords:** Indigenous, Access, Healthcare, Nicaragua, Rama, Cultural Safety

## Abstract

Indigenous Peoples worldwide experience significantly worse health outcomes than people who are non-Indigenous. The challenges they face are generally more severe in low- and middle-income countries, but most research has focused on high-income countries, mainly Australia, Canada, New Zealand and the USA. This study aimed to help redress this imbalance by focusing on access to healthcare for the Rama indigenous people, a community of around 2,000 people who live on the Caribbean Coast of Nicaragua. The data for this study were drawn from face-to-face and online interviews with Rama and non-Rama participants, conducted during two periods: one in spring 2016 and one in January 2024. In total, 49 participants were interviewed, 28 of whom were female. The research found that although government healthcare was nominally free, limited supplies, equipment shortages, and staff constraints pushed many Rama people to rely on expensive private care. This could strain household incomes, which were already under greater pressure than other local, non-indigenous households. Travel to tertiary care centres was costly and difficult because of the distance between Rama communities and where most regional healthcare centres were based. This was one contributory factor that Rama families often took into account when making decisions about when and where to seek healthcare. The language barrier between Rama service users and non-Rama health professionals, perceived discrimination, and non-Rama health professionals’ lack of local knowledge further discouraged care-seeking; and there were many barriers for Rama people who wished to train as health professionals. Although Traditional Medicine was valued, its use declined between 2016 and 2024. Progress towards culturally safe healthcare for the Rama will be difficult; supporting more Rama people to become medically trained and promoting longer-term placements in Rama communities for trained health professionals could help in its achievement.

## Background

Globally, Indigenous Peoples experience much poorer health outcomes than those who are non-Indigenous [30, 51, 89, 90]. The importance of improving the health of Indigenous Peoples has long been recognised by multiple national and international bodies. In 1989, the International Labour Organization Convention (No. 169) declared a right to health services for Indigenous Peoples and called for individual government responsibility to provide community-based, culturally-appropriate care [21]. The Pan American Health Organization (PAHO) passed two resolutions (CD37.R5 and CD40.R6) during the mid 1990s that further sought to promote the right to health and access to healthcare for Indigenous Peoples and urged member governments to “allow for representation of Indigenous Peoples in the development of healthcare services for their own populations” [21, p. 3]. The United Nations declared the first International Decade of the World’s Indigenous People, which ran 1995–2004, and was partly aimed at addressing the disparity in health outcomes between Indigenous Peoples and those who are non-Indigenous. Because the goals of the first decade were not met, the United Nations chose to adopt a second International Decade of the World’s Indigenous People (2005–2014) [89, 97]. The United Nations Declaration on the Rights of Indigenous Peoples (UNDRIP) followed in 2007, Article 24 of which affirmed “the rights of Indigenous Peoples to their traditional medicines and health practices, and to all social and health services” [98, p. 8].

However, even following these initiatives, and numerous national programmes aimed at reducing the health gap between Indigenous Peoples and those are who non-Indigenous [75] – including cultural awareness-style training [65] and health promotion interventions [59, 69] – poor health outcomes for Indigenous Peoples have remained [36, 80]. Key health indicators, such as child nutrition and infant and maternal mortality, remain poorer for Indigenous Peoples than those who are non-Indigenous [36, 109]. The life expectancy of Indigenous Peoples is up to 20 years shorter than the majority population in some countries [96, 99, 108, 109]. In some settings, the prevalence of chronic diseases – including diabetes, heart disease and high blood pressure – is higher amongst Indigenous Peoples than the majority population [2, 95]. The inequality between Indigenous Peoples and people who are non-Indigenous was also seen very clearly during the Covid-19 pandemic, during which Indigenous Peoples had a higher risk of contracting Covid-19 and a higher mortality risk than those who are non-Indigenous [54].

There have been many reasons given for why Indigenous Peoples have poorer health outcomes than those who are non-Indigenous. For example, Indigenous Peoples are more likely to live in poverty or be of lower socio-economic status than the rest of the national population, more likely to experience worse living conditions and have a worse diet, and less able to afford healthcare [39, 89, 90]. They are also more likely to be adversely affected by societal transitions, such as dietary shifts and land-use changes, because of their greater reliance on livelihood strategies based on their connection to natural resources [10, 39]. Relatedly, many Indigenous Peoples are involved in conflict over natural resources, which can also contribute towards poorer health outcomes [10, 74]. These latter two factors are only liable to become more pronounced in the future because of climate change and the increased development and marketisation of Indigenous communities [8].

### Access to healthcare

Being able to access health services is an important determinant of good health for Indigenous Peoples [35]. The concept of ‘access’ is not, however, uniformly defined [32]. Much previous research has focused exclusively on physical access [40], which was determined based on measures such as the number of households within a particular radius of a health facility. This ignored other important considerations such as the condition of roads, topography and other natural barriers [77].

Donabedian et al. [33] argued that access to healthcare should be based not just on the presence of a health facility, but also whether the population is able to use it. The work of Thomas and Penchansky [94] extended the concept of healthcare access to consider personal, financial and organisational barriers to service utilisation. Thaddeus and Maine [92] focused on different phases of delay in seeking healthcare for maternal care, noting factors including: the perceived severity of the illness; who is making the decision to seek care and for whom; and the adequacy of the healthcare destination in terms of its referral procedures and availability of supplies, equipment and trained personnel. This latter factor points towards later scholarship that saw access to healthcare as a negotiated property between service users and health professionals [20, 32, 75]. Scholarship since the work of Thaddeus and Maine [92] has tried and classify all the different factors related to access to healthcare. For example, Levesque et al. [52] conceptualised access to healthcare as stemming from 10 different complementary dimensions and personal abilities, including the affordability and acceptability of services [52].

There are many factors that can determine access to healthcare for Indigenous Peoples. For example, it is common for health services to be located further away from where Indigenous Peoples live and those that are nearby tend to be of lower quality [6, 22, 53]. In most countries, mainstream healthcare services are not designed with the healthcare needs of Indigenous populations at the forefront [49]. In relation to access to healthcare being a negotiated property, an important barrier for many Indigenous Peoples is that they do not speak, or have lower comprehension of the language of the majority population in their country [49], which may mean they struggle to communicate with non-Indigenous healthcare workers. Indigenous Peoples often experience racism or discrimination in healthcare settings [34, 43, 70], and this can contribute towards trauma, poorer mental health, internalised oppression, and a desire to avoid formalised healthcare providers [35, 49, 51]. These experiences, along with the loss or weakening of Indigenous identities in many communities is also linked to higher levels of suicide risk, alcohol use and substance use [16, 23, 87].

### Heterogeneity of Indigenous Peoples

Indigenous Peoples have been defined at the group level as those who have a different identity and culture from the national society, do not have political representation at a national level, may be linguistically distinct from the national society, and base their livelihoods on local resources [90]. The definition of “Indigenous Peoples” is, however, largely for the benefit of international agreements: there is great heterogeneity amongst Indigenous Peoples, with recent estimates suggesting there are 476 million Indigenous Peoples, representing around 5,000 different cultures, and living across 90 countries [35, 84, 108]. Healthcare challenges across these different cultures are, thus, not universal, as there are many different healthcare needs, different views of healthcare, and varied notions of wellness across these different cultures [56, 84]. As with all populations, there is also notable within-group heterogeneity: numerous studies have shown how Indigenous women face greater difficulties when accessing healthcare than do Indigenous men [42, 46, 82].

Access to healthcare for Indigenous Peoples living in High-Income Countries (HICs) is different than it is for those living in Low- and Middle-Income Countries (LMICs), with many of the barriers to healthcare affecting Indigenous Peoples in HICs being more pronounced in LMICs. A literature review by Morgan et al. [64] found that: Indigenous Peoples in LMICs have less purchasing power for medicines, treatments, and food and accommodation (if needed); medications are often not subsidised or freely available; healthcare facilities tend to be poorer, with medical equipment often being worse and/or more unreliable; and there is often insufficient funding to maintain staff availability.

### Rationale for the study

The evidence base on access to healthcare for Indigenous Peoples in LMICs is limited, with most research having been conducted in HICs, primarily in Australia, Canada, New Zealand and the USA [31, 36, 39, 75]. In fact, even national- and local-level disaggregated data on important health indicators – such as infant mortality, life expectancy at birth or principal causes of morbidity and mortality – can be hard to obtain for Indigenous Peoples living in some LMICs [67]. Clearly then, more research is needed to document Indigenous experiences of accessing healthcare in LMICs. This study aimed to contribute towards this by investigating the barriers to healthcare access for the Rama indigenous people of Nicaragua.

### Case study: Nicaragua

**Fig. 1:**
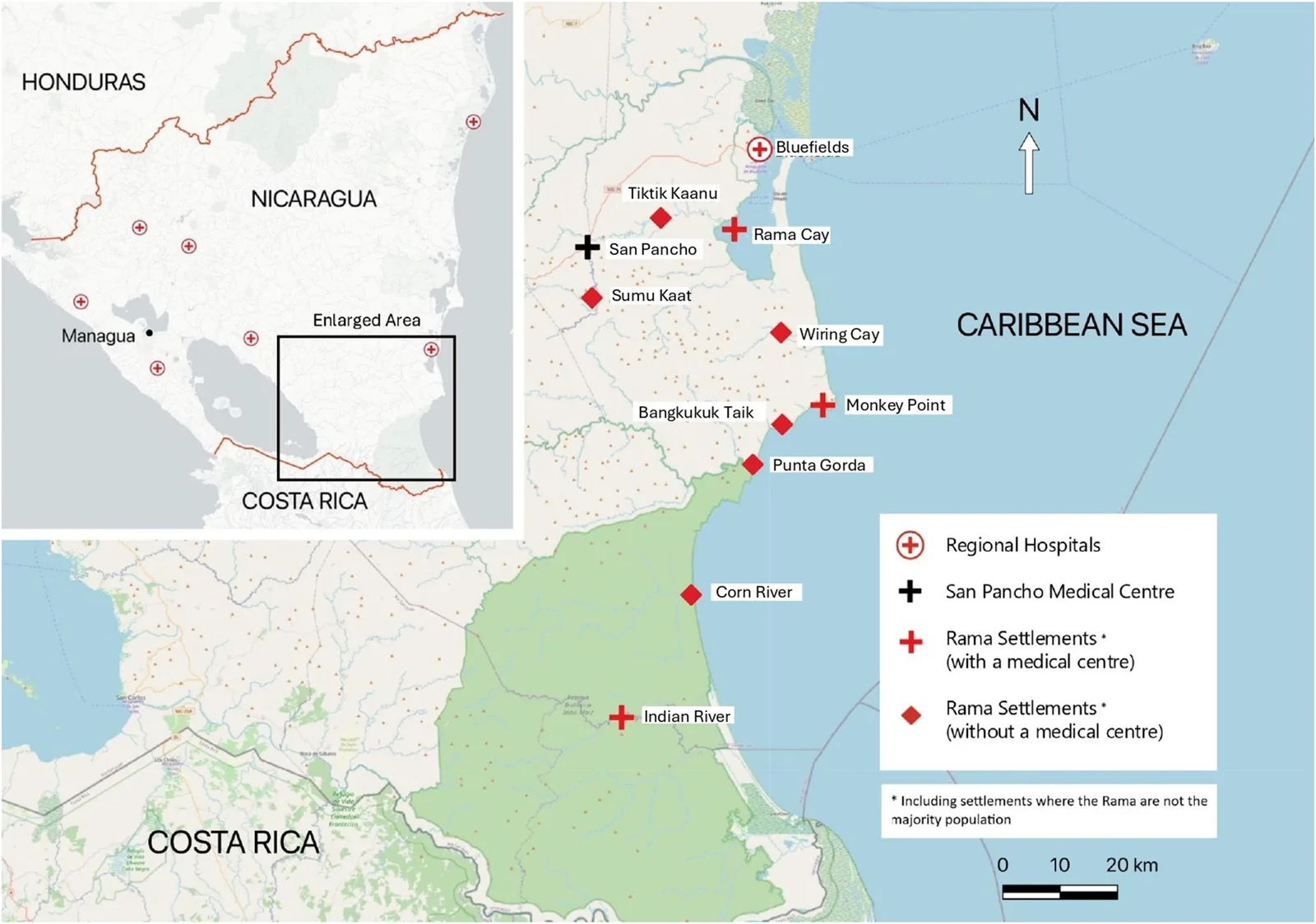
Healthcare in the Rama communities

The Rama indigenous people live in the Southern Autonomous Region of the Caribbean Coast of Nicaragua (La Región Autónoma de la Costa Caribe Sur – RACCS). They number approximately 2,000 people (compared to the entire Nicaraguan population of c. 6.9 m). Across the nine communities where Rama people live, there are three small health centres: one on the island of Rama Cay, and one each in the communities of Monkey Point and Rio Indio (see Fig. 1). There is also a medical centre in the non-Rama town of San Pancho, which is located within the Rama-Kriol territory, and the regional hospital is based in the nearby city of Bluefields, the capital of the RACCS. There has been very little recent research on the health of the Rama: two studies focused on health and identity for all people in the RACCS were published in the early 1990s [12, 13]; and three studies were published on Rama midwifery and ethnobotany in 2008, but based on data collection from the early and mid 1990s [26–28]. In addition, Rama Cay was one of the data collection sites for a study published in 2012, which was focused on the acceptability of Clean Delivery Kits across the RACCS [62].

Nicaragua has a free, universal healthcare system based on the Community-Family Health Model [60], which aims to bring healthcare as close as possible to where people live and work. Health professionals in rural areas rotate through providing healthcare in different communities, as do unpaid volunteer “Brigadistas” – trained by the Nicaraguan Ministry of Health – who reside in the communities they serve for short periods [1]. A 2008 report by the Program for Appropriate Technology in Health found several challenges facing the Nicaraguan health system, including: a shortage of health personnel and high caseloads; poor healthcare provision in rural areas; and poor data sharing [86]. The country has made considerable progress in health outcomes since then. Life expectancy at birth in 2024 was 75 years, 8.7 years higher than it was in 2000; and many other health indicators, including maternal mortality and HIV infection incidence rate, have also improved [71].

The Caribbean Coast of Nicaragua, however, is considerably poorer than the rest of the country [61]. Its population also has a different demographic profile than the country at large, with those who live on the Caribbean Coast (collectively called Costeños) belonging to six different ethnic groups [7]. The Coast was not formally settled by the Spanish before the country gained its independence, but instead became first an English, then British protectorate, and was then exploited by US multi-national corporations [55, 81]. After the Nicaraguan revolution ended in 1979, the victorious Sandinista Government introduced a series of social reforms, including literacy programmes [15]. Whilst these were very successful for the country overall, this marked a change for Costeños in comparison to the previous administration’s *laissez-faire* approach [63]. Over time, mistrust between the Sandinistas and Costeños increased. During the Nicaraguan Civil War in the 1980s, the US-backed counter-revolutionary forces, who fought the Sandinistas, were drawn disproportionately from Costeños. The Caribbean Coast was granted autonomy from the national government in 1987, but due to Government neglect of the principles of autonomy, insufficient investment, land and resource conflicts, and other factors, this has failed to secure effective self-determination for Costeños [7, 24, 38, 88].

The Nicaraguan constitution states its support for the Indigenous Peoples of Nicaragua to “maintain and cultivate their own cultural identities [and] manage their local affairs within their traditions and historical customs” [21, p. 3]. Nicaragua’s General Health Law, introduced in 2005, called for Indigenous Peoples on the Caribbean Coast to be supported to “develop health methods that are consistent with their traditions and communities”, and Law 759, introduced in 2011, was designed to specifically address “equity and access to traditional and ancestral medicine” [21, p. 4]. Despite these written decrees, however, the realisation of true equity and access to healthcare for Nicaragua’s Indigenous people has proven to be difficult [21].

### Cultural Safety

The concept of Cultural Safety was originally developed by Maori nurses in New Zealand, proposed as an approach to help alleviate some of the factors that can affect Indigenous access to healthcare [65, 66, 107]. Its aims are to shift away from healthcare approaches that are based on service users’ ethnic backgrounds and towards more reflection on the provider’s approach; consider the power imbalances between Indigenous service users and non-Indigenous healthcare providers during consultations; and focus on patient agency [66, 107]. Cultural Safety can, therefore, be conceptualised as lying on a continuum from Cultural Awareness at one end, moving through Cultural Sensitivity and Cultural Competence, to Cultural Safety at the other. The emphasis in Cultural Awareness is on the health professional knowing more *about* the patients’ culture, whereas the focus of Cultural Safety is on the relationship between the health professional and the patient—a move from a “them,” through “them and us,” to an “us” understanding [100]. Cultural Safety is defined by the recipient of care, not the provider.

Culturally safe healthcare, therefore, requires the reorientation of health services so they are more able to meet the needs of vulnerable groups, through engagement with the intersectional relationships – based on race, poverty, gender, and other factors – that exist between service users and health professionals [19]. Using this lens, healthcare is understood as a form of social relationship whereby people (service users and health professionals) negotiate access to resources [50, 91], which reinforces the point that access to healthcare includes both the availability of services and the delivery of services [20, 32, 57, 92].

Some recent scholarship has contended that whilst Cultural Safety can be defined by the patient’s experience of healthcare (as mentioned), it must also be linked to an improvement in health outcomes for Indigenous Peoples [65]. To this end, researchers have argued that Cultural Safety can be a useful framework to consider policy aims, including: the creation of healthcare spaces that are safe, inviting and socially accepting; national health strategies that are inclusive of Indigenous Peoples; and a decolonisation of nursing education and practice [19, 20, 36, 45, 57, 85, 91]. The vast majority of Cultural Safety research to date has, however, taken place in HICs. Two recent reviews on the concept only included one article not set in Australia, Canada, New Zealand or the US, despite synthesising 89 articles between them (four articles were used in both reviews) [5, 29]. By focusing on Nicaragua, this study will contribute towards appraisals of the concept for LMICs.

### Research questions

This study will investigate the following research questions:

1. What were Rama perceptions of the healthcare services available to them?
2. Which factors did the Rama believe affected their ability to access these healthcare services?
3. To what extent did Rama experiences of healthcare align with, or diverge from culturally-safe healthcare?

## Methods

**Fig. 2:**
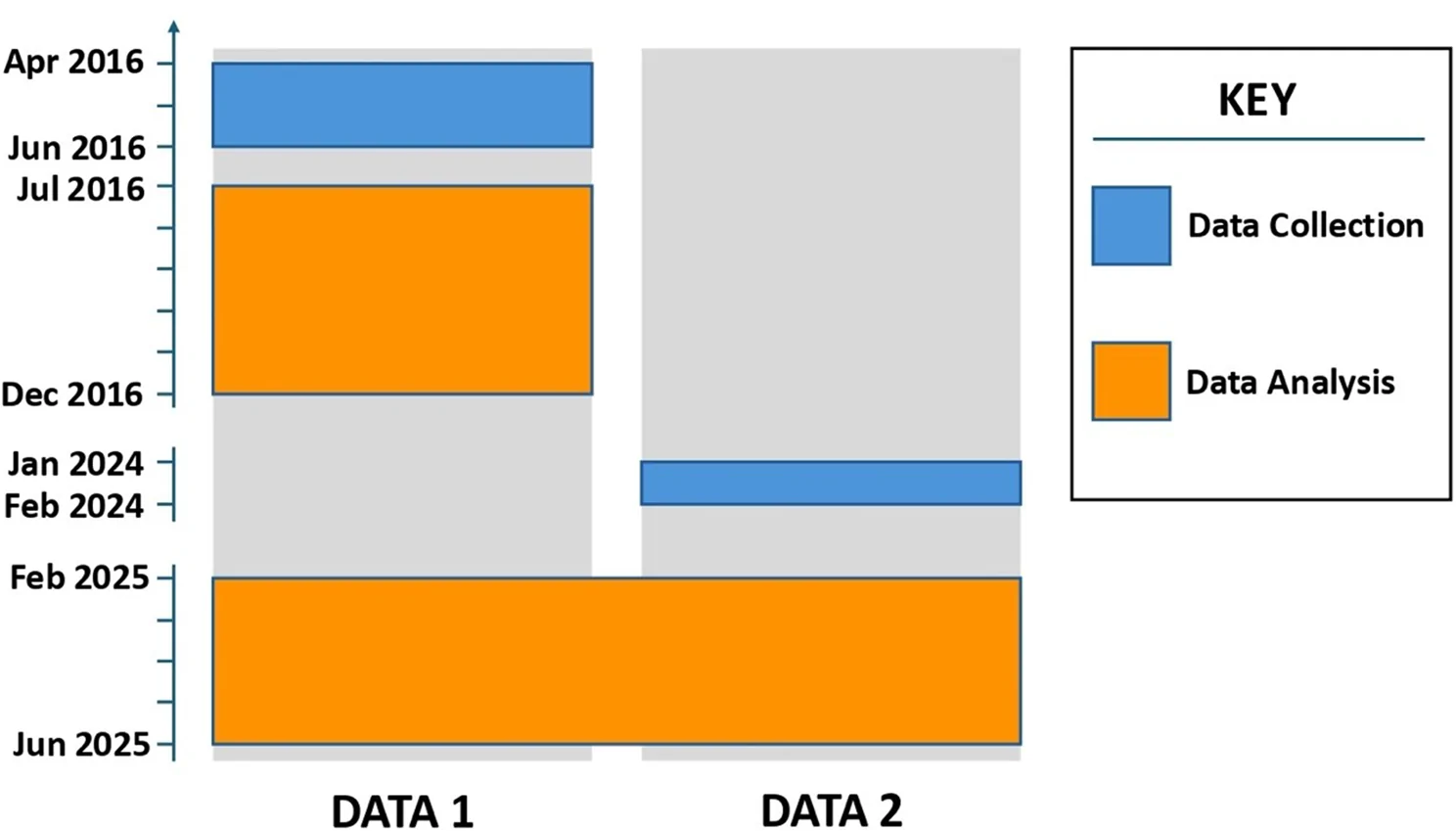
Data collection and analysis timeline

We took a pragmatic approach to data collection for this study, with interviews conducted with Rama and non-Rama participants between February and May 2016, and in January 2024 (see Fig. 2). This data collection was nested within two other larger, ethnographic studies [8–10]. The researchers included one male, British academic, with experience of conducting mixed-methods research with vulnerable populations; one male, Rama community member, with experience of facilitating research projects; and one female, Nicaraguan Creole research assistant (2016 only), with experience of working on community-based research.

The semi-structured interviews conducted in 2016 were designed specifically to focus on access to healthcare, with the intent to present the findings as a standalone study. A short report of the results was shared with local stakeholders, and workshops were held with Rama service users in two Rama communities (Rama Cay and Sumu Kaat). In these workshops, attendees commented on findings, helped shape the analysis, and received gifts of food as requested by Rama community leaders. Due to time constraints and the priorities of funders, however, the findings were not written up and published in a peer-reviewed journal.

The field trip that took place in 2024 for the second larger study presented an opportunity to augment the data from 2016, make comparisons between the two periods, and focus on important new areas of enquiry, such as the impact of the Covid-19 pandemic. This was deemed especially important given the paucity of research on the health of this population. The main impetus for the collection of the 2024 data, however, came from the participants themselves—in many cases, the interview conversations conducted for the larger study touched on access to healthcare in an unstructured way. This difference in the type of data collected (largely semi-structured interviews in 2016 and largely unstructured interviews in 2024) led to a slightly different approach to analysis for the two sets of data (see below).

### Sampling and recruitment

In 2016, the study population was drawn from all individuals (18+) who had a lived experience of access to healthcare in the Rama communities. This was designed to include Rama service users (as the focus of enquiry), health professionals, and other community members. The intent in 2024 was to collect data for the larger ethnographic study being conducted at the same time, but many participants in the Rama communities interviewed wanted to also speak about access to healthcare.

Participants were recruited in a largely convenience-based sample from five locations in the northern part of the RACCS: the Rama settlements of the island of Rama Cay (where the majority of the Rama population lives) and the villages of Tiktik Kaanu and Sumu Kaat (which are small, farming-focused communities further inland); and two major, non-Rama settlements, the city of Bluefields and the town of San Pancho. In the case of the Rama communities, participants were generally recruited by visiting every house in turn. In this instance, the research was usually introduced to potential participants by the lead author (AP), who was already known to potential participants because of the activities of the larger studies (interviews and observations in 2016; mapping and observations in 2024), and/or because he was living (2016) or based (2024) in the study locations at the time. Some participants also directly approached the research team during the ethnographic fieldwork being conducted for either of the two larger studies. Other participants – typically the health professionals and other community members included in the study – were recruited using snowball sampling via personal and professional contacts built up in Bluefields during the time spent in the region. Three non-Rama health professionals who were working on Rama Cay in 2016 were also invited to participate.

Potential participants were either given a participant information sheet or talked through the sheet in their preferred language, then given the opportunity to ask questions. AP explained that their participation in the research was voluntary and that they could withdraw at any time. Potential participants were told that all the research team would be able to see what they had said, but that GW (because of his role within the community) would not know who had said what. They were further assured that anything they said would not be discussed outside the research team and that it would not be possible to identify them in anything that was written or presented to others based on the research. Potential participants generally had a period of at least 24 hours to consider taking part, unless they decided they wanted to participate immediately. Informed consent was taken before the interview started.

### The interviews

Interviews were conducted by AP and took place in people’s homes, or the researcher and participants chose a space located away from other people in a noisy public building or space, or the corner of a cafe or restaurant, where the conversation could not be easily overheard. Risk assessments were completed before the data was collected and AP was in regular contact with supervisors/managers at his respective home institutions throughout the data collection period. AP worked closely with GW and other Rama community leaders to try and mitigate the power imbalance inherent in the researcher-participant relationship. For example, the female research assistant was present in interviews in 2016 if requested, and participants were able to take part in the interviews in pairs if they wished.

The interviews in 2016 used a topic guide that was developed with researchers with experience of conducting research in Nicaragua and piloted with Rama community members in January 2016. As mentioned above, the interviews in 2024 were less structured, with the interviews framed more by what the participants wanted to speak about. However, the preliminary analytical themes developed in 2016 (see below), and the original topic guide from 2016, were used to help formulate follow-up questions.

The interviews were recorded on an encrypted audio device, or handwritten notes were made if participants did not want to be recorded. These notes did not include participants’ names and were destroyed as soon as electronic copies were created and stored on an encrypted device. All data were stored in accordance with Data Management Plans that were completed at the home institutions for the lead author.

All interviews were conducted in Spanish or Rama Creole, the latter of which has many similarities to Standard English. The quotes included in the results section have been transliterated into English or translated from Spanish into English, and then back translated from English to Spanish to check for errors, following established best practice [61, 72, 73]. Interviews were either recorded, or if the participant did not wish this to happen, detailed contemporaneous notes were made. All interview recordings were transcribed verbatim by the authors. Participants were given gifts of food to compensate them for their time, as requested by Rama community leaders.

### Data analysis

Analysis took place over two time periods (see Fig. 2) and was led by AP, but with both authors discussing the findings regularly. Transcripts were analysed alongside the detailed, contemporaneous notes of the non-recorded interviews. The data from 2016 were first coded deductively [83], with this drawing upon feedback received from the workshops with Rama service users, and the Levesque framework, which divides access to healthcare into five dimensions: 1) Approachability; 2) Acceptability; 3) Availability and Accommodation; 4) Affordability; and 5) Appropriateness [52].

A meeting was held with participants after the data was collected in 2024 during which preliminary findings were shared and discussed. Because of the more unstructured nature of the data collected in 2024, a narrative approach was used initially [11, 14], which led to the development of individual, case-study accounts that spoke to the core issues already highlighted. The research team then created additional case studies from the 2016 data, before using the 2024 to flesh out the original deductive themes with the new data. In 2024, the decision was made to reorient the study towards Cultural Safety and so the subsequent analysis was more rooted by this concept, with the themes reviewed against core concepts including power, identity and trust. Across our analysis, similarities and differences within and between the deductive codes and the narrative accounts were interrogated. The study thus used a pluralistic approach to data analysis, resulting in the combination of themes and illustrative case studies (see below), which has been used in other health-focused research studies [93, 110].

## Results

Across the two fieldwork periods, 49 participants were interviewed, 28 of whom were female (Table 1). Of these participants, seven were health professionals (of mixed gender), comprising: a Rama nurse who had worked across the region before retiring to Rama Cay, a Rama trainee nurse; a Traditional Medicine practictioner based on Rama Cay; two non-Rama nurses from Bluefields who were working and living on Rama Cay; a non-Rama community nurse based in Bluefields; and a *Brigadista* doctor who was attending patients on Rama Cay during the first data collection period. Six of the participants (including two health professionals) were interviewed in pairs in three interviews (all in 2016). Data analysis resulted in five themes and four illustrative case studies (Table 2).

**Table 1:** Demographics of participants

	2016	2024	Totals
Respondents	31	18	49
No. female	21	7	28
Health professionals	6	1	7
Non-Rama	4	0	0
Age range (average)	22–76 (45.8)	18–64 (37.2)	18–76 (42.7)

**Table 2:** Themes and case studies

	Themes and sub-themes	Case Study
Theme 1:	Availability and distance • Health services in Rama settlements • Access to tertiary care • Healthcare-seeking behaviour	Case Study 1: Rama Man (#24.16)
Theme 2:	Cost of healthcare	Case study 2: Rama Woman (#16.7) Case Study 3: Three respondents
Theme 3:	Healthcare provision	
Theme 4:	Relationship between service users and health professionals	Case Study 4: Four respondents
Theme 5:	Traditional medicine vs Western medicine • Declining use of Traditional medicine	

### Availability and distance

#### Health services in Rama settlements

Participants talked about the state-funded health provision that was available in Rama settlements, which saw no change between 2016 and 2024. From a regional perspective, health services are designed to prioritise the needs of pregnant women, children under five years old and the elderly. There were health centres located in three Rama settlements (see also, Fig. 1), with the largest situated on Rama Cay. The utility of these was severely constrained, however, as they all had restricted opening hours—for example, the Rama Cay health centre was supposed to be open 8–12 and 2–4, Monday to Friday, but it was usually not actually staffed (#16.15).

There was also a reliance on transient staff—a non-Rama health professional explained that all health professionals rotated around different communities in the RACCS, usually spending 6–12 months in each placement (#16.6). Volunteer Brigadistas might also be in attendance every two weeks or so, depending on their commitments across the region (Participant #16.6). Occasionally, specific health initiatives meant that doctors and other health professionals from the Pacific coast of Nicaragua visited Rama communities for short periods. One of these visits took place during the fieldwork period in 2016. Whilst there was, therefore, a basic level of clinical presence, in practice these factors combined meant that healthcare within the Rama communities themselves was often precarious. The communities either only had local professional support for a few hours a day, excluding weekends, or had none at all. This “patchy” localised care forced the Rama to rely on tertiary facilities in Bluefields or Managua for any serious medical needs. The relationship between patients and health professionals, and how this aspect of the current healthcare provision for the Rama is undermining Cultural Safety, is outlined in great detail below (see Theme 4).

#### Access to tertiary care

The transition to tertiary care was not merely a matter of distance, but of temporal uncertainty. For serious illnesses, Rama people in the northern part of the Rama-Kriol territory usually needed to travel to the regional capital, Bluefields, or the national capital, Managua. Getting to Bluefields quickly required fast boat transportation, but this was often difficult to arrange.

*We call up to Bluefields and them take 30 minutes to almost one hour […] so if it be serious, still it could be getting almost two hours to Bluefields. Rama Woman #16.4*

This participant (above) was referring to the free and fast Government-funded boat, but this was frequently in high demand and could be located in a community a long way from where it was needed. If this was the case, there might not be transportation available for many hours—a critical delay for acute conditions, which could be fatal. Two non-Rama health professionals based on Rama Cay in 2016 (#16.11 and #16.12) gave an example of when they had called for an emergency boat for a patient at midday, but it had only arrived at 5pm, by which time the patient had already passed away.

Each Rama settlement had its own transport-based barriers to accessing tertiary healthcare. For example, those living in Monkey Point (see Fig. 1) faced an even longer boat journey in an emergency of between an hour (using the fast Government boat) to one-and-a-half hours in a standard motor-powered boat (#24.14).

In Sumu Kaat in 2024, access depended on where participants lived within the community. Those living in the centre of the community or its eastern side were required to travel first to the health centre in San Pancho by boat or horseback (depending on the weather), both of which would take approximately three hours, and then onwards by road to Bluefields.

*There is some complication to go to San Pancho because it not every day there be a [boat]. And it hard, you see, [going by] horse when it dry. Rama Man (#24.04)*

For those living in the western side of the community, however, the new main road built between Managua and Bluefields could be reached on foot in 40 minutes, from where an ambulance could meet the patient and take them to Bluefields (#24.8 and #24.9).

This illustrates one change between the two fieldwork periods: mobile phone reception was much improved in 2024. Poor communication was a frustration for health professionals and Rama people alike in 2016; whereas in 2024, the Rama could more easily call ahead in an emergency. The common theme across participants from all the different Rama communities represented in both fieldwork periods, however, was that someone with a healthcare emergency was likely to face a long journey (in time) to a tertiary healthcare facility.

#### Healthcare-seeking behaviour

In part because of how long it could take to access tertiary healthcare, and because of the shortcomings of the health provision in Rama communities (see Theme 3), the decision of whether to travel to a healthcare facility was preceded by a multi-day observation period where the Rama might wait to see how severe an ailment was before travelling to where they could receive the next level of healthcare.

*We be far from San Pancho, [so] some time we take maybe two days, three days [when] we watching the sick, how is it coming on. When you go to more sicker, harder, then we take [the person] to San Pancho [then] when you real sick bad, they send you to Bluefields in ambulance. Rama Man (#24.08)*

Sometimes this had meant the decision to seek healthcare came too late. One Rama woman (#16.7) talked about two different women with cancer, both of whom did not travel to Managua to receive treatment until they were very sick. This delay meant that by the time they arrived at a hospital with the right facilities for their needs, it was too late to provide curative care.

In other cases, the decisions made about where to obtain suitable treatment could be reliant on several factors, including the availability and standard of care (see Case Study 1).

### Case study 1: emergency care

**Table:** 

*One participant, “Eulogico” talked about the time he was bitten by a snake when planting crops close to where he lived. His uncle practiced Traditional Medicine (see Theme 5), and knew how to produce a suitable antidote, but his uncle was away in another community at the time. As the other community was still closer than travelling to Bluefields, however, the decision was made to transfer Eulogico there. It was not possible to contact his uncle ahead of time, however, so when Eulogico arrived, his uncle then had to go and find the right plants to produce the right medicine. The medicine was effective, but it had been administered so late that Eulogico was now gravely unwell and had to be transferred first to Bluefields and then on to Managua for treatment before making a full, but slow recovery.* Rama Man (#24.16)	

### Cost of healthcare

Despite the existence of free, universal healthcare in Nicaragua, hidden costs are an important barrier to access. Transportation acts as the first economic gatekeeper for the Rama when accessing healthcare. For example, participants said that if the Government-funded boat was unavailable, they would have to pay for transportation. One participant (#24.06) said this could cost about 2500C$ (c. US$69), which compares to a teacher’s average monthly salary of 5500C$ (US$149).

For non-emergency care, Rama people often chose to access private healthcare or specialists because they were viewed as being more effective and because the Rama health centres often didn’t have the right equipment (#16.1 and #16.17) (see also Theme 3). Prescription costs were covered when Rama people used the Rama health centres, but these often did not have the correct medicines, at which point they would have to travel to pharmacies elsewhere and pay high prices (#16.1 and #16.4).

When receiving healthcare in Bluefields, or in a health centre that wasn’t near to where they lived, Rama people also had to pay for their ancillary costs – food and everything else they needed for the duration of their treatment (#16.6, #16.19 and #16.30). These added costs could exceed a household’s total income and therefore have a large impact on family finances. In some cases, families had sold essential food supplies or took out loans, effectively trading their food and financial security for immediate medical intervention (see Case Studies 2 and 3).

Rama people who worked in Government jobs, such as teachers, usually had all their healthcare costs covered.

*I am […] retired from my work in education [and so] I not pay nothing for this operation. Rama Man (#16.2)*

Whilst indigenous status often dictates general healthcare access, these data suggest there were within-group disparities where specific socioeconomic advantages, such as government employment, allowed some Rama people to bypass the systemic failures of local health centres. Evidence of this inequality supports previous research that has revealed the socioeconomic diversity within the Rama community [8], and highlights the need for health services to account for this so as to protect those who are most vulnerable.

### Case study 2

**Table:** 

*One participant’s mother, “Maria”, had been diagnosed with cancer. Maria had to travel to Bluefields on a regular basis and her medicines cost C$5,000 (US$138) per month, which was more than her family’s total monthly income. Maria’s daughter said she and her siblings took out loans to help pay for these costs. They also ate cheaper food and sold all the fish and prawns they had caught in the Bluefields Lagoon instead of including it in their diets.* Rama Woman (#16.7)	

### Case study 3

**Table:** 

*One Rama family had a baby who was severely undernourished. The baby’s mother, “Shanda”, was breastfeeding both the baby and an older sibling, but both she and her husband were malnourished themselves. The doctor in Bluefields suggested a dietary supplement, but it cost C$220 (US$5.50) every three weeks, which was not affordable for them. Shanda said she was trying hard to raise the money to pay for the supplement and was following the doctor’s other recommendations, but she and her husband could not find work. They had to cut down on food for their other children and had to stop buying other essential items.* Non-Rama Health Professionals (#16.11; #16.12) Rama Woman (#16.15)	

### Healthcare provision

Participants said that an important barrier to effective healthcare was the deficiencies in healthcare facilities. One non-Rama health professional (#16.6) said that each Municipality^1^ should have a hospital, laboratory, midwifery centre, mental healthcare unit, and children’s department. However, the participants’ descriptions of healthcare facilities in the Rama-Kriol territory revealed a significant gap between governmental policy and reality, with Rama healthcare centres lacking basic utilities.

Two non-Rama health professionals based on Rama Cay at the time of the first fieldwork period (#16.11 and #16.12) spoke at length about the limitations of the health centre they worked in. There was no electricity in the health centre, which meant that any emergency out-of-hours care had to be conducted using torches. This also meant the health centre did not have a fridge, so all vaccinations and medicines had to be administered on the day they arrived from Bluefields or they could not be used, creating a “feast or famine” cycle of medicine provision. Other health professionals confirmed that the situation was the same in other Rama communities (#16.6 and 16.25).

Reflecting some of the attitudes expressed about individual health professionals (see Theme 4), some Rama service users viewed the poor local facilities as a form of deliberate institutional neglect, which eroded their trust in the ability of the healthcare system to care for them.

*If you arrive there with a hard pain, they don’t have equipment for operations or tests. Rama Woman (#16.7)*

*You see with our health centre here they not got some equipment […] Them equipment for them use for us, for strong accident, sickness, I see like them not got them. I gone to [Bluefields] and beg them for this we need, but them not bring them [to our health centre]. Rama Man (#16.13)*

### Relationship between service users and health professionals

Access to healthcare was further complicated by a profound socio-cultural disconnect between Rama service users and non-Rama health professionals. Although the rotation system for staffing meant many Rama communities were staffed by trained health professionals when they might not otherwise have been, some Rama participants said the non-Rama health professionals often lacked local knowledge and/or did not have the time to get to know their patients.

*Of course, the problem is to get a permanent doctor. It sometime now [since] we have permanent doctor and that be better for knowing we. Rama Man (#16.2)*

Some Rama perceived non-Rama staff as being dissatisfied with their rural postings, viewing these as a financial burden rather than a service, which meant they often had poor attitudes towards Rama service users.

*They act this way because it’s not them that is feeling that good about here. [But] they are getting their salary off of we, so why they not be happy? Rama Woman (#16.21)*

Some participants felt that this dissatisfaction with rural life was also a key reason why so few non-Rama health professionals completed their full rotation when posted in Rama settlements (#16.16 and #16.21). These views were supported by two non-Rama health professionals working on Rama Cay in 2016 who expressed dissatisfaction with their postings, saying that their rent, transport and food costs were so much more than those of their peers who were working elsewhere (#16.11 and #16.12). Neither of them ended up completing their full rotation.

Secure and long-term placements that can engender trust and understanding between health professionals and their patients can help to develop Cultural Safety [107]. It is also important for health professionals to be able to reflect on their own practice [29], which they are unlikely to be able to do during short-term placements. For these two reasons, the system of staff rotation, and the poor retention of staff in posts embedded in Rama communities, was fundamentally undermining the development of Cultural Safety for the Rama.

The lack of Cultural Safety in patient-health professional relationships was also visible in linguistic exclusion: non-Rama health professionals usually did not use the same first language (Spanish) as their Rama patients (Rama Creole). This not only affected relationships but also came with the risk of potential misdiagnoses.

*Some of we Rama here from Sumu Kaat, some of we don’t know how to talk Spanish, you know? When Spaniard doctor say, “What you feeling?” maybe you can’t tell him what you get. Rama Man (#24.08)*

*From Bluefields they send a doctor to stay here for like 11 months, but […] they don’t know how to speak on our people because our people don’t all know how to speak Spanish, so [the Rama] have difficulty to express their feelings and what them feel. Rama Man (#24.02)*

Past experiences were very powerful in framing perceptions about whether care was culturally safe or not. Many Rama specifically talked about when they had travelled to larger hubs like Bluefields or San Pancho and had been treated poorly or were ignored by non-Rama health professionals.

*In Bluefields [and] in that side, they not treat those Rama so well. The Pacific side, the Spaniard […] sometime they don’t attend you, you have to go to the help, and sometimes they help you and sometime they don’t. Rama Man (#24.13)*

*Sometimes the doctors tend to the people bad and they don’t want to go back. Rama Woman (#16.3)*

*The Spaniard don’t want to attend you good. You have to wait a whole day until they attend to you. Rama Woman (#24.06)*

Gendered cultural frictions were highlighted by two non-Rama health professionals interviewed (#16.11 and #16.12) who believed the influence of “machismo”^2^ culture, which is widespread amongst all Nicaraguan ethnic groups [37], affected the care of many Rama woman. They gave the example of one woman who had been diagnosed with ovarian cancer and whose husband refused to let her have an operation because “she wouldn’t be a real woman afterwards.” A focus on Cultural Safety in this instance highlights the importance of shared decision making between patients and health professionals, as well as the need for health professionals, or allied professionals, who can navigate within Rama social structures with sensitivity.

Most Rama participants said that they would prefer to be seen by healthcare professionals who were Rama.

*Rama doctor be all better […] Me say that they maybe attend you more quicker and better because it be our own people […] But sure, they understand you better. [The non-Rama health professionals] not understand you like we understand. Rama Man (#24.04)*

While the Rama expressed a clear preference for Rama healthcare providers, it was not easy for Rama people to become qualified health professionals because of the cost of training. On top of course fees, students had to buy their own equipment and live in Bluefields during the course of their studies, both of which were very expensive for Rama people. One participant discussed the difficulties his daughter had faced when trying to train as a nurse: she had had to pause her studies to take up manual work because she couldn’t afford the right clothing and equipment.

*Right now, my daughter [is] working because [she needs to buy] shoes, you need clothes. She had to stop studying [and will have to return] next year, because she need money for these things and for practice, the equipment. [Now] she hauling rock [working in construction]. Rama Woman (#16.7)*

A key tenet of Cultural Safety is that the healthcare workforce should reflect the population it serves [107] It is difficult to argue this was the case for the Rama when there were so few Rama health professionals working in the Rama communities during the two data collection periods.

### Traditional medicine vs Western medicine

Many participants said that Traditional Medicine was very important for the Rama. The Rama called this “Bush Medicine”, which was juxtaposed with Western, “White” or “Doctor” Medicine. This medical pluralism was driven by both personal preference and pragmatic necessity, as well as by perceptions of their effectiveness and relative cost—the former often being seen as more effective for specific ailments and/or more affordable.

*I not like go [to the] bush doctor. Them not have apparatus to look in you to know what it is you got. Rama Man (#16.31)*

*People love to use [Traditional Medicine] here in Rama Cay because it’s more effective than [Western medicine] and it’s cheaper. Rama Man (#24.02)*

*One of my niece, she did got a bad sore and she did attend that doctor over to the next side at the health centre, and they did her a treatment […] and that bad sore never cure. [So] she just gone and go look the bush, different bush and mix it up and she boil it. And she washed the sore, the bad sore [and] the next day […] it cured it. Rama Man (#16.13)*

The ways that this medical plurality operated through heterogeneous attitudes within the Rama community was displayed through the discussions with participants held in 2024 about the impact of the Covid-19 pandemic on the Rama (see Case Study 4).

### Case study 4: the Covid-19 pandemic

**Table:** 

*The response of many Rama people to Covid-19 was to avoid large population centres, including Bluefields and San Pancho. Many Rama people turned moved towards the use of Traditional Medicine because Western medicine was seen as ‘not working’. When the vaccine became available in Spring 2022, many Rama people did not trust it, thinking it would harm them without providing any real benefit. The first vaccines to reach some of the more remote Rama communities were administered to Rama people by non-Rama health professionals from Bluefields. Brigadistas also attended community events, including baseball games, to try increase vaccination coverage.* Rama Men (#24.09; #24.13; #24.01) Rama Woman (#24.14)	

#### Declining use of traditional medicine

The use of traditional medicine was in a state of rapid decline for the Rama, which was very notable between the two fieldwork periods. In 2016, it was difficult to determine exactly how many Rama people practised Traditional Medicine.

*We have many people that is bush doctor. I couldn’t tell you how many, but they say that two man [here] we have is bush doctor. Two people over the other side [too, and then] we have two boy more here that could help. We have plenty if you want to be attend. Rama Woman (#16.10)*

In contrast, in 2024 there appeared to be fewer Traditional Medicine practitioners.

*The one bush doctor, him cover all nine (Rama) communities and moves around different places, so it is quicker to go to San Pancho now [for Western medicine instead]. Rama Woman (#24.14)*

This decline appeared to be at least partly the result of increased deforestation of the Rama-Kriol territory by Pacific Nicaraguans to facilitate cattle ranching, with one Traditional Medicine practitioner (Participant #16.24) saying that this had reduced the availability and accessibility of the raw materials needed for Traditional Medicine.

Another possible explanation given was that fewer younger people were interested in becoming Traditional Medicine practitioners—this view was shared by two participants in 2024 and the interview with a Traditional Medicine practitioner in 2016 also appeared to support this:

*He said he has not passed it on because nobody is interested in learning from him. He feels he is important for the community, but nobody wants to learn and nobody in the community helps him to go and get the medicines. Fieldwork notes: Interview with Rama Health Professional (#16.24)*

The loss of these practitioners, combined with the ongoing structural barriers to Western medicine, left the Rama in a precarious position with both their traditional safety net and the state-sponsored system increasingly out of reach.

## Discussion

This study examined fieldwork data from 2016 and 2024 to investigate the factors that affect the ability of the Rama indigenous people to access healthcare.

### Summary of findings

There were two health centres located in Rama communities in the northern part of the Rama-Kriol territory, but both of these offered a limited service. Other facilities included a medical centre in the town of San Pancho – which is a non-Rama town located within the Rama-Kriol territory – and the regional hospital based in the city of Bluefields. Travel to these latter two medical facilities could be difficult and expensive, so Rama people often took a phased approach to their health seeking, waiting to see how serious an ailment was before deciding where to go.

Although the cost of some healthcare was covered by the Government, there was often insufficient equipment, medical supplies and expertise available in medical facilities, meaning that the Rama people often used private healthcare. The cost of this and/or other expenses related to accessing healthcare, including prescriptions and accommodation costs, often had an impact on families’ livelihoods.

Working in the medical facilities based in Rama communities was not always popular amongst non-Rama healthcare professionals. Some Rama people felt less able to communicate well with non-Rama health professionals because of language barriers and because they believed the non-Rama health professionals had insufficient local knowledge. Some Rama people felt they were not treated well by non-Rama health professionals. It was not easy for the Rama to become health professionals themselves.

Some Rama people had had bad previous experiences of healthcare services or did not believe in the effectiveness of the care they received. Traditional Medicine was seen as an important alternative to ‘Western’ medicine, but its use was much diminished between the two data collection periods (2016 and 2024).

### Contribution to the literature

This study’s results support similar research that has found Indigenous Peoples often live further away from health facilities than people who are non-Indigenous and, as a result, may delay seeking healthcare. For example, a literature review, drawing upon published research from Africa, Asia and the Americas, found that Indigenous women were most likely to attend ‘doorstep’ antenatal services and only accessed health facilities further away if there were complications during labour [3]. Thaddeus and Maine [92] argued that the obstacles encountered throughout the healthcare system are intertwined, feeding back into individual decision making. Thus, under the circumstances that might exist for the Rama – including knowing that there is little that can be done for a patient because medical facilities are inadequate – the decision not to seek healthcare is reasonable [92].

An important finding from this study was that the cost of transport or lengthy stays away from home to receive healthcare could have longer impacts on the livelihoods of individual Rama families. Whilst Nicaragua’s system of socialised medicine was a positive for many Rama – especially for those who worked, or had previously worked in Government roles – it could not alleviate the more systemic issues related to facilities, equipment and medicine supply. Many participants said they felt forced to buy medications from private organisations or to seek care from alternative, private medical centres. This has also been seen in research conducted in other settings, including Guatemala, Bangladesh and with other Indigenous groups in Nicaragua [21, 47, 68].

Existing research suggests that Indigenous women, children and elderly people are particularly affected by poor healthcare provision [18, 48, 64, 76]. From this perspective, it is positive that the Nicaraguan healthcare system is designed to attend to the needs of these three groups first. The results of this study, however, suggest that some attitudes towards Rama women bore similarities to that found in other similar research, specifically the need for some women to seek approval from their families, particularly husbands, before receiving healthcare [4].

Mental health and non-communicable diseases were not a strong feature of the findings of this study. Whilst cancer was mentioned by participants, some of the conditions that have been notable amongst studies conducted in similar contexts, such as diabetes, were not [68]. It seems unlikely that this is because there is less awareness of these conditions in Nicaragua—although the incidence of cancer in Nicaragua is lower than many of its regional neighbours and the world in general [17, 101–104], Nicaragua’s incidence of NCDs overall is higher than the world average [105, 106]. It is possible that the incidence of these conditions is much lower amongst the Rama in comparison to other Nicaraguans, but this is unknown because of the lack of disaggregated data available within the country. The lack of reference to mental health facilities suggests that further research in this area is required.

Policies such as the placement of health professionals in rural areas have been recommended as ways to improve Indigenous healthcare [44, 54]. The way the Nicaraguan health system supports this initiative and its use of Brigadistas could, therefore, be seen as a positive. The data presented here, however, suggest that the constant rotation of staff has meant there are few long-term, trusted relationships between health professionals and their Rama patients, which is an important principle of Cultural Safety. Many Rama participants commented that many non-Rama health professionals had a poor understanding of local issues. Many also commented on the issue of the language barrier and others said they thought Rama people were actively discriminated against by non-Rama health professionals.

Despite the importance of Indigenous involvement in healthcare being emphasised in Nicaraguan Government initiatives and laws in a way that could assist with the aims of Cultural Safety, there has been little success seen on the ground [21]. For example, for the achievement of Cultural Safety, healthcare workforce should reflect the population they serve as closely as possible [107], but this is not currently the case in the Rama communities. In order for this to improve, there needs to be more support for Rama people to train as health professionals, an approach that has been endorsed in other settings [4]. Any attempt to achieve this, however, will require many barriers to be removed, most notably the prohibitive cost of studying at university for most Rama. Initiatives to reduce upfront costs for those least able to pay, such as through the free provision of training equipment, or fee waivers for those who go on to work for a certain time in rural and/or Indigenous communities could be explored. These are likely to prove difficult, however, as the Nicaraguan Government has recently banned all foreign NGOs from the country, which have often been the main source of funding and management for similar schemes. The requirement for health professionals to speak Spanish may continue to be a barrier for some Rama people. In the meantime, however, the introduction of Cultural Safety Navigators – individuals who are able to be cultural brokers and interpreters between Rama patients and non-Rama health professionals – would be a useful preliminary step [79].

Another important factor for the implementation of Cultural Safety is ensuring that Indigenous perspectives are integrated into the top-level structures and management of a country’s health system. The focus of Government-led initiatives in Nicaragua in this vein has been the integration of Traditional Medicine and ‘Western’ medicine [21, 58]. This seems to have been already taking place in some manner in the case of the Rama, perhaps without any formal assistance, with many participants said they used a mixture of Bush and Western medicine. The evidence from other countries supporting policies of this nature is positive: research in Nigeria and Ghana showed that shared-care delivered by traditional and faith healers alongside primary health-care providers was effective at improving mental health outcomes [41]. It was noticeable from the interviews with Rama participants, however, that the use of Traditional Medicine appeared to have declined considerably between 2016 and 2024. This was possibly because young people were not interested in studying Traditional Medicine, but also because increased cattle ranching within the Rama-Kriol territory had reduced the availability of plants required for the production of medicines [10].

A focus on Cultural Safety cannot solve all the factors affected the Rama’s access to healthcare, including the lack of funding available, and the wider and entrenched geographical inequalities that exist in the country. Nicaraguan health professionals, whether Rama or non-Rama, are often under financial pressure and are seeking to provide the best healthcare they can without necessary equipment and supplies. Even the most culturally competent doctor cannot overcome the lack of a refrigerator for vaccines or five-hour boat delays. For the Rama, true healthcare access requires a dual approach: a commitment to the relational principles of Cultural Safety and a significant investment in the physical and economic infrastructure that currently leaves them isolated.

Equally, Cultural Safety is based on the premise of the decolonisation of healthcare, but Nicaraguan majority structures are bound up in their own, unique post-colonial and post-imperial histories. There is also a long and complex history of relationships between the present Government and the Indigenous Peoples who live on the Caribbean coast, stretching back to the end of the Nicaraguan revolution in 1979 [7, 81]. Moreover, Government initiatives have often been beset by problems and the Caribbean Coast has not been a priority of central government officials since the granting of autonomy to the Caribbean Coast regions in 1987 [24, 38].

Overall then, it is reasonable to argue that the concept of Cultural Safety will be difficult to achieve in the RACCS in Nicaragua. Nevertheless, the principles of Cultural Safety point towards clear, interim policy solutions: increasing the length of time that health professionals spend in each placement, which could lead to longer-term, trusting relationships between healthcare professionals and their patients; the introduction of Cultural Safety Navigators; supporting more Rama people to become medically trained; and the embedding of indigenous leaders into regional decision making.

In terms of next steps, more research on the healthcare of the Rama is important as more information about the existing challenges in all the Rama communities, and how these might be alleviated, is needed. Future research could consider using participatory approaches, which have been shown to be able to develop feasible and well-accepted interventions to improve healthcare [25, 78]. There is also a need for more research investigating the concept of Cultural Safety in LMIC settings, in order to continue rebalancing the literature, which is currently dominated by research conducted in HIC settings.

### Strengths and weaknesses

One strength of this study was its use of qualitative methods, which enabled the collection of rich insights on access to healthcare for the Rama. Collecting data from two time periods helped to increase the number of study participants and meant that the findings could be compared for similarities and differences over time. Another important strength of the study was that most of the participants were women, which contrasts with most previous research with this population. This was no doubt greatly facilitated by the research assistant who was part of the project during the data collection period in 2016.

Although safeguards were put in place to lessen the impact of Rama community leaders being involved in data collection, some participants may have felt unable to share everything they wanted to in the knowledge that someone in their community may see what they had said. Participants were drawn from all the Rama communities in the northern part of the Rama-Kriol territory, which provided important representation of different perspectives, but future research should also seek to include the views of those living in the southern part of the Rama-Kriol territory. Participants were drawn from varied populations, but no healthcare managers or commissioners were interviewed. It will be important to include these perspectives in future research so as to more fully understand the importance of policy and funding on barriers to healthcare access.

## Conclusion

This study suggests the Rama, like many Indigenous people, face numerous barriers when seeking to access healthcare. Many of these are due to distance, transport costs, and systemic inequities. Even though Nicaragua has socialised healthcare, shortages of equipment, medicine, and staff push many Rama to seek costly private care. Policies such as rural health placements and the work of the Brigadistas are positive, and have been championed by other academics, but Rama participants still reported poorer care than non-Rama people. Reasons for this included language barriers, perceived discrimination, and a perception that non-Rama health professionals did not have long-lasting relationships with their Rama patients and lacked cultural understanding when responding to them. Increasing the number of Rama health professionals is important, but has been hindered by high education costs. Some integration of Traditional and Western medicine exists, but Traditional Medicine is declining due to several factors, including youth disinterest and environmental damage from cattle ranching. Non-communicable diseases and mental health issues were rarely mentioned by participants, suggesting gaps in awareness or service provision that require further research.

A focus on Cultural Safety could potentially help alleviate some of the barriers affecting the Rama’s access to healthcare. Unlike “cultural competence,” which focuses on the clinician’s knowledge, Cultural Safety centres on the patient’s experience of power, respect, and identity, and can serve as a critical defence for improving access by addressing the relational and linguistic barriers that currently alienate Rama service users. However, Cultural Safety alone cannot overcome all factors contributing to the Rama’s access to healthcare, such as the country’s weak institutions, underfunded services, entrenched inequalities, and a complex history between the state and Indigenous communities.

## Data Availability

The study followed the same principles for data storage as those followed for the two larger studies. The datasets analysed during the current study are available from the corresponding author upon reasonable request.

## Footnotes

1 Nicaragua has 15 Departments and two Autonomous Regions, one of which is the RACCS where the Rama live. Each of these 17 local government areas are further subdivided into Municipalities, of which there are 153 across the whole country. Almost the entire Rama-Kriol territory is contained within the Bluefields Municipality.

2 Defined as the cultural concept of manliness, which includes the importance of male dominance and hypermasculine traits such as aggression. Machismo may disempower women and foster sexist attitudes.

## References

[1] Adamo M, Rivera D, Shah R, Koepsell J, Martínez L, Ortiz JP, et al. Time volunteered on community health activities by brigadistas in Nicaragua. Rev Panam Salud Publica. 2016;40(5):388–95. 28076589

[2] AIHW. Aboriginal and torres strait islander health performance framework - summary report. 2025. https://www.indigenoushpf.gov.au/reports/summary-reports/summary-report.

[3] Akter S, Davies K, Rich JL, Inder KJ. Indigenous women’s access to maternal healthcare services in lower- and middle-income countries: a systematic integrative review. Int J Public Health. 2019;64(3):343–53. 10.1007/s00038-018-1177-4. 30506363

[4] Akter S, Davies K, Rich JL, Inder KJ. Community perspectives of barriers indigenous women face in accessing maternal health care services in the Chittagong Hill Tracts, Bangladesh. Ethn Health. 2022;27(5):1222–40. 10.1080/13557858.2020.1862766. 33356512

[5] Allice I, Acai A, Ferdossifard A, Wekerle C, Kimber M. Indigenous cultural safety in recognizing and responding to family violence: a systematic scoping review. Int J Environ Res Public Health. 2022;19(24):16967. 10.3390/ijerph192416967. 36554846 PMC9779508

[6] Australian Institute of Health and Welfare. Remoteness and the health of indigenous australians. 2014. https://www.aihw.gov.au/getmedia/3fae0eb7-b2be-4ffc-9903-a414388af557/7_7-indigenous-health-remoteness.pdf.aspx.

[7] Papworth A. An analysis of the food security of the Rama indigenous group, Nicaragua. Unpublished PhD Thesis. University College London; 2019. https://discovery.ucl.ac.uk/id/eprint/10069791/.

[8] Papworth AJ, Maslin M, Randalls S. The challenges of a food sovereignty perspective: an analysis of the foodways of the Rama indigenous group, Nicaragua. Food Sec. 2022;14:1013–26. 10.1007/s12571-022-01268-x.

[9] Papworth A, Maslin M, Randalls S. How food-system resilience is undermined by the weather: the case of the Rama Indigenous group, Nicaragua. Ecol Soc. 2022. 10.5751/ES-13376-270401.

[10] Papworth A and Windell G. Mapping as resistance: land grabs and illegal deforestation in the Rama indigenous territory, Nicaragua. Presentation to the The RGS-IBG Annual International Conference; 2024. https://pure.york.ac.uk/portal/en/publications/mapping-as-resistance-land-grabs-and-illegal-deforestation-in-the/.

[11] Bamberg M. Narrative analysis: an integrative approach. In: Järvinen M, Mik-Meyer N, editors. Qualitative analysis: eight approaches for the social sciences. Sage; 2020.

[12] Barrett B. Health care behavior on Nicaragua’s Atlantic coast. Soc Sci Med. 1993;37(3):355–68. 10.1016/0277-9536(93)90266-7. 8356484

[13] Barrett B. Ethnomedical interactions: health and identity on Nicaragua’s Atlantic coast. Soc Sci Med. 1995;40(12):1611–21. 10.1016/0277-9536(94)00348-W. 7660174

[14] Bengtsson T, Andersen D. Narrative analysis: thematic, structural and performative. In: Järvinen M, Mik-Meyer N, editors. Qualitative analysis: eight approaches for the social sciences. Sage; 2020.

[15] Bourgois P. Class, ethnicity, and the state among the Miskitu Amerindians of northeastern Nicaragua. Lat Am Perspect. 1981;8(2):22–39. 10.1177/0094582X8100800204.

[16] Brady M. Culture in treatment, culture as treatment. A critical appraisal of developments in addictions programs for indigenous North Americans and Australians. Soc Sci Med. 1995;41(11):1487–98. 10.1016/0277-9536(95)00055-C. 8607039

[17] Bray F, Laversanne M, Sung H, Ferlay J, Siegel RL, Soerjomataram I, et al. Global cancer statistics 2022: GLOBOCAN estimates of incidence and mortality worldwide for 36 cancers in 185 countries. CA Cancer J Clin. 2024;74(3):229–63. 10.3322/caac.21834. 38572751

[18] Brown K, Clarke M. First Nations women’s health 2023. Aust NZ J Obst Gynaeco. 2023;63(3):275–77. 10.1111/ajo.13687. 37340604

[19] Browne AJ, Smye VL, Rodney P, Tang SY, Mussell B, O’Neil J. Access to primary care from the perspective of aboriginal patients at an urban emergency department. Qual Health Res. 2011;21(3):333–48. 10.1177/1049732310385824. 21075979

[20] Cameron BL, Carmargo Plazas MDP, Salas AS, Bourque Bearskin RL, Hungler K. Understanding inequalities in access to health care services for aboriginal people: a call for nursing action. Adv Nurs Sci. 2014;37(3):E1–16. 10.1097/ANS.0000000000000039. https://journals.lww.com/advancesinnursingscience/fulltext/2014/07000/understanding_inequalities_in_access_to_health.13.aspx. 25102218

[21] Carrie H, Mackey TK, Laird SN. Integrating traditional indigenous medicine and western biomedicine into health systems: a review of Nicaraguan health policies and miskitu health services. Int J Equity Health. 2015;14(1):129. 10.1186/s12939-015-0260-1. 26616532 PMC4663733

[22] Chan J, Friborg J, Zubizarreta E, van Eck JW, Hanna TP, Bourque J-M, et al. Examining geographic accessibility to radiotherapy in Canada and Greenland for indigenous populations: measuring inequities to inform solutions. Radiother Oncol. 2020;146:1–8. 10.1016/j.radonc.2020.01.023. 32065874

[23] Chandler MJ, Lalonde C. Cultural Continuity as a Hedge against Suicide in Canada’s first Nations. Transcult Psychiatry. 1998;35(2):191–219. 10.1177/136346159803500202.

[24] Chapman C. One of Nicaragua’s greatest achievements is in danger of being reversed. Minority Rights Group. 2007. https://minorityrights.org/one-of-nicaraguas-greatest-achievements-is-in-danger-of-being-reversed/.

[25] Chomat AM, Menchú AI, Andersson N, Ramirez-Zea M, Pedersen D, Bleile A, et al. Women’s circles as a culturally safe psychosocial intervention in Guatemalan indigenous communities: a community-led pilot randomised trial. BMC Womens Health. 2019;19(1):53. 10.1186/s12905-019-0744-z. 30943958 PMC6448212

[26] Coe FG. Ethnobotany of the Rama of Southeastern Nicaragua and comparisons with Miskitu plant lore1. Econ Bot. 2008;62(1):40–59. 10.1007/s12231-008-9006-y.

[27] Coe FG. Ethnomedicine of the Rama of Southeastern Nicaragua. J Ethnobiol. 2008;28(1):1–38. 10.2993/0278-0771_2008_28_1_eotros_2.0.co_2.

[28] Coe FG. Rama midwifery in eastern Nicaragua. J Ethnopharmacol. 2008;117(1):136–57. 10.1016/j.jep.2008.01.027. 18337033

[29] Curtis E, Jones R, Tipene-Leach D, Walker C, Loring B, Paine S-J, et al. Why cultural safety rather than cultural competency is required to achieve health equity: a literature review and recommended definition. Int J Equity Health. 2019;18(1):174. 10.1186/s12939-019-1082-3. 31727076 PMC6857221

[30] Curtis E, Wikaire E, Stokes K, Reid P. Addressing indigenous health workforce inequities: a literature review exploring ‘best’ practice for recruitment into tertiary health programmes. Int J Equity Health. 2012;11(1):13. 10.1186/1475-9276-11-13. 22416784 PMC3402985

[31] Davy C, Harfield S, McArthur A, Munn Z, Brown A. Access to primary health care services for Indigenous peoples: a framework synthesis. Int J Equity Health. 2016;15(1):163. 10.1186/s12939-016-0450-5. 27716235 PMC5045584

[32] Dixon-Woods M, Cavers D, Agarwal S, Annandale E, Arthur A, Harvey J, et al. Conducting a critical interpretive synthesis of the literature on access to healthcare by vulnerable groups. BMC Med Res Methodol. 2006;6(1):35. 10.1186/1471-2288-6-35. 16872487 PMC1559637

[33] Donabedian A, Wheeler JRC, Wyszewianski L. Quality, cost, and health: an integrative model. Med Care. 1982;20(10):975–92. 10.1097/00005650-198210000-00001. 6813605

[34] Durey A, Thompson SC, Wood M. Time to bring down the twin towers in poor Aboriginal hospital care: addressing institutional racism and misunderstandings in communication. Intern Med J. 2012;42(1):17–22. 10.1111/j.1445-5994.2011.02628.x. 22032537

[35] Durie M. Providing health services to indigenous peoples. Vol. 327. British Medical Journal Publishing Group; 2003. p. 408–09. 10.1136/bmj.327.7412.408 PMC188483 12933709

[36] Ferdinand A, Lambert M, Trad L, Pedrana L, Paradies Y, Kelaher M. Indigenous engagement in health: lessons from Brazil, Chile, Australia and New Zealand. Int J Equity Health. 2020;19(1):47. 10.1186/s12939-020-1149-1. 32731870 PMC7393707

[37] Gámez NL, Guevara Fletes ADLÁ, Núñez JA, Espinoza Solís R. Incidencia del patrón de conducta machista en la violencia intrafamiliar en una colonia de la ciudad de León. 2024.

[38] González M. The unmaking of self-determination: twenty-five years of regional autonomy in Nicaragua. Bull Latin Am Res. 2016;35(3):306–21. 10.1111/blar.12487.

[39] Gracey M, King M. Indigenous health part 1: determinants and disease patterns. Lancet. 2009;374(9683):65–75. 10.1016/S0140-6736(09)60914-4. 19577695

[40] Gulliford M, Figueroa-Munoz J, Morgan M, Hughes D, Gibson B, Beech R, et al. What does ‘access to health care’ mean? J Health Serv Res Policy. 2002;7(3):186–88. 10.1258/135581902760082517. 12171751

[41] Gureje O, Appiah-Poku J, Bello T, Kola L, Araya R, Chisholm D, et al. Effect of collaborative care between traditional and faith healers and primary health-care workers on psychosis outcomes in Nigeria and Ghana (COSIMPO): a cluster randomised controlled trial. Lancet. 2020;396(10251):612–22. 10.1016/S0140-6736(20)30634-6. 32861306 PMC8473710

[42] Harris A, Zhou Y, Liao H, Barclay L, Zeng W, Gao Y. Challenges to maternal health care utilization among ethnic minority women in a resource-poor region of Sichuan Province, China. Health Policy Plan. 2010;25(4):311–18. 10.1093/heapol/czp062. 20100776

[43] Harris RB, Cormack DM, Stanley J. Experience of racism and associations with unmet need and healthcare satisfaction: the 2011/12 adult New Zealand Health Survey. Aust N Z J Public Health. 2019;43(1):75–80. 10.1111/1753-6405.12835. 30296819

[44] Health Council of Canada. Empathy, dignity, and respect: creating cultural safety for Aboriginal people in urban health care. In: Health Council of Canada Ottawa, Ontario, Canada. 2012.

[45] Horrill T, McMillan DE, Schultz ASH, Thompson G. Understanding access to healthcare among Indigenous peoples: a comparative analysis of biomedical and postcolonial perspectives. Nurs Inq. 2018;25(3):e12237. 10.1111/nin.12237. PMC6055798 29575412

[46] Ibáñez-Cuevas M, Heredia-Pi IB, Meneses-Navarro S, Pelcastre-Villafuerte B, González-Block MA. Labor and delivery service use: indigenous women’s preference and the health sector response in the Chiapas Highlands of Mexico. Int J Equity Health. 2015;14(1):156. 10.1186/s12939-015-0289-1. 26698570 PMC4688940

[47] Islam A. Health system in Bangladesh: challenges and opportunities. Am J Health Res. 2014;2(6):366–74. 10.11648/j.ajhr.20140206.18.

[48] Jongen C, Campbell S, Saunders V, Askew D, Spurling G, Gueorguiev E, et al. Wellbeing and mental health interventions for Indigenous children and youth: a systematic scoping review. Child Youth Serv Rev. 2023;145:106790. 10.1016/j.childyouth.2022.106790.

[49] King M, Smith A, Gracey M. Indigenous health part 2: the underlying causes of the health gap. Lancet. 2009;374(9683):76–85. 10.1016/S0140-6736(09)60827-8. 19577696

[50] Kirkham SR, Baumbusch JL, Schultz ASH, Anderson JM. Knowledge development and evidence-based practice: insights and opportunities from a postcolonial feminist perspective for transformative nursing practice. Adv Nurs Sci. 2007;30(1):26–40. 10.1097/00012272-200701000-00004. https://journals.lww.com/advancesinnursingscience/fulltext/2007/01000/knowledge_development_and_evidence_based_practice_.4.aspx. 17299282

[51] Kirmayer LJ, Brass G. Addressing global health disparities among Indigenous peoples. Lancet. 2016;388(10040):105–06. 10.1016/S0140-6736(16)30194-5. 27108233

[52] Levesque J-F, Harris MF, Russell G. Patient-centred access to health care: conceptualising access at the interface of health systems and populations. Int J Equity Health. 2013;12(1):18. 10.1186/1475-9276-12-18. 23496984 PMC3610159

[53] Liao Z-Y, Kean S, Haycock-Stuart E. Indigenous lands and health access: the influence of a sense of place on disparities in post-stroke recovery in Taiwan. Health Place. 2024;86:103210. 10.1016/j.healthplace.2024.103210. 38354468

[54] Little BB, Shakib S, Pena Reyes ME, Karimi S, Vu GT, Dupré N, et al. COVID-19 infection and mortality among non-pregnant indigenous adults in Mexico 2020-2022: impact of marginalisation. J Glob Health. 2023;13:06030. 10.7189/jogh.13.06030. 37506193 PMC10386760

[55] Loveland C. Rural-urban dynamics: the miskito Coast of Nicaragua. Urban Anthropol. 1973;182–93.

[56] McBeath BM. Conceptualization of community wellness in three first nations communities. Queen’s University (Canada); 2020.

[57] McGibbon E, Etowa J, McPherson C. Health-care access as a social determinant of health. Can Nurse. 2008;104(7):22–27. 18856224

[58] Mendoz Galan MJ, Gordillo Tobar A, Herrera E, Rosdriguez E. Nicaragua: integration of western and indigenous traditional medicine. 2017.

[59] Mikhailovich K, Morrison P, Arabena K. Evaluating Australian indigenous community health promotion initiatives: a selective review. Rural Remote Health. 2007;7(2):1–18. 10.22605/RRH746. 17550334

[60] MINSA. Marco Conceptual Modelo de Salud Familiar y Comunitario. 2008.

[61] Mitchell EM, Steeves R, Dillingham R. Cruise ships and bush medicine: globalization on the Atlantic Coast of Nicaragua and effects on the health of creole women. Public Health Nurs. 2015;32(3):237–45. 10.1111/phn.12127. 24766610 PMC4211993

[62] Mitchell EM, Steeves R. The acceptability of clean delivery kits on the Atlantic Coast of Nicaragua: a focused ethnography. Hisp Hlth Care Int. 2012;10(1):36–41. 10.1891/1540-4153.10.1.36. PMC3512193 23226103

[63] Moore JH. The Miskitu national question in Nicaragua: background to a misunderstanding. Sci Soc. 1986;50(2):132–47. 10.1177/003682378605000202.

[64] Morgan, Breau, M, Breau M. Access to maternal health services for Indigenous women in low- and middle-income countries: an updated integrative review of the literature from 2018 to 2023. Rural Remote Health. 2024;24(2):8520. 10.22605/rrh8520. 38826130

[65] Muise GM. Enabling cultural safety in Indigenous primary healthcare. Healthc Manage Forum. 2019;32(1):25–31. 10.1177/0840470418794204. 30304957

[66] Nelson SE, Wilson K. Understanding barriers to health care access through cultural safety and ethical space: indigenous people’s experiences in Prince George, Canada. Soc Sci Med. 2018;218:21–27. 10.1016/j.socscimed.2018.09.017. 30316132

[67] Nettleton C, Napolitano D, Stephens C. An overview of current knowledge of the social determinants of indigenous health (Working paper). 2007.

[68] Nieblas-Bedolla E, Bream KDW, Rollins A, Barg FK. Ongoing challenges in access to diabetes care among the indigenous population: perspectives of individuals living in rural Guatemala. Int J Equity Health. 2019;18(1):180. 10.1186/s12939-019-1086-z. 31752908 PMC6873569

[69] Oosman S, Nisbet C, Smith L, Abonyi S. Health promotion interventions supporting Indigenous healthy ageing: a scoping review. Int J Circumpolar Health. 2021;80(1):1950391. 10.1080/22423982.2021.1950391. 34313553 PMC8317950

[70] Ortiz MS, Baeza-Rivera MJ, Salinas-Onate N, Flynn P, Betancourt H. Healthcare mistreatment attributed to discrimination among mapuche patients and discontinuation of diabetes care. Rev Med Chil. 2016;144(10):1270–76. 28074982 10.4067/S0034-98872016001000006

[71] PAHO & WHO. Nicaragua - country profile. 2024. https://hia.paho.org/en/country-profiles/nicaragua.

[72] Papworth A, Maslin M, Randalls S. The challenges of a food sovereignty perspective: an analysis of the foodways of the Rama Indigenous group, Nicaragua. Food Sec. 2022;14(4):1013–26. 10.1007/s12571-022-01268-x.

[73] Papworth A, Maslin M, Randalls S. How food-system resilience is undermined by the weather: the case of the Rama Indigenous group, Nicaragua. Ecol Soc. 2022;27(4):Article 1. 10.5751/ES-13376-270401.

[74] Pedersen D. Reframing political violence and mental health outcomes: outlining a research and action agenda for Latin America and the Caribbean region. Cien Saude Colet. 2006;11(2):293–302. 10.1590/S1413-81232006000200008.

[75] Peiris D, Brown A, Cass A. Addressing inequities in access to quality health care for indigenous people. Can Med Assoc J. 2008;179(10):985. 10.1503/cmaj.081445. 18981431 PMC2572646

[76] Pelcastre-Villafuerte BE, Meneses-Navarro S, Ruelas-González MG, Reyes-Morales H, Amaya-Castellanos A, Taboada A. Aging in rural, indigenous communities: an intercultural and participatory healthcare approach in Mexico. Ethn Health. 2017;22(6):610–30. 10.1080/13557858.2016.1246417. 27788597

[77] Perry B, Gesler W. Physical access to primary health care in Andean Bolivia. Soc Sci Med. 2000;50(9):1177–88. 10.1016/S0277-9536(99)00364-0. 10728839

[78] Prost A, Colbourn T, Seward N, Azad K, Coomarasamy A, Copas A, et al. Women’s groups practising participatory learning and action to improve maternal and newborn health in low-resource settings: a systematic review and meta-analysis. Lancet. 2013;381(9879):1736–46. 10.1016/s0140-6736(13)60685-6. 23683640 PMC3797417

[79] Rankin A, Baumann A, Downey B, Valaitis R, Montour A, Mandy P. The role of the indigenous patient navigator: a scoping review. Can J Nurs Res. 2022;54(2):199–210. 10.1177/08445621211066765. 35014886 PMC9109580

[80] Riecken T, Scott T, Tanaka MT. Community and culture as foundations for resilience: participatory health research with first nations student filmmakers. Int J Multiling Indigenous Health. 2006;3(1):7–14.

[81] Riverstone G. Living in the land of our ancestors: Rama Indian and Creole Territory in Carribbean Nicaragua. ASDI; 2004.

[82] Ruiz MJ, van Dijk MG, Berdichevsky K, Munguía A, Burks C, García SG. Barriers to the use of maternity waiting homes in indigenous regions of Guatemala: a study of users’ and community members’ perceptions. Cult Health Sex. 2013;15(2):205–18. 10.1080/13691058.2012.751128. 23234509

[83] Saldaña J. The coding manual for qualitative researchers. 4th ed. Sage; 2021.

[84] Sarivaara E, Maatta K, Uusiautti S. Who is indigenous? Definitions of indigeneity. Eur Sci J. 2013.

[85] Sarmiento I, Paredes-Solís S, Dion A, Silver H, Vargas E, Cruz P, et al. Maternal health and Indigenous traditional midwives in southern Mexico: contextualisation of a scoping review. BMJ Open. 2021;11(12):e054542. 10.1136/bmjopen-2021-054542. PMC8710897 34949629

[86] Sequeira M, Espinoza H, Amador JJ, Domingo G, Quintanilla M, de Los Santos T. The Nicaraguan health system. Seattle, WA: PATH. 2011;201(1). 10.2471/BLT.11.086124 PMC3150763 21836761

[87] Skewes MC, Blume AW. Understanding the link between racial trauma and substance use among American Indians. 2019. 10.1037/amp0000331. PMC6338088 30652902

[88] Sollis P. The Atlantic Coast of Nicaragua: development and autonomy. J Lat Am Stud. 1989;21(3):481–520. 10.1017/S0022216X00018526. http://www.jstor.org/stable/156960.

[89] Stephens C, Nettleton C, Porter J, Willis R, Clark S. Indigenous peoples’ health—why are they behind everyone, everywhere? Lancet. 2005;366(9479):10–13. 10.1016/S0140-6736(05)66801-8. 15993213

[90] Stephens C, Porter J, Nettleton C, Willis R. Disappearing, displaced, and undervalued: a call to action for Indigenous health worldwide. Lancet. 2006;367(9527):2019–28. 10.1016/S0140-6736(06)68892-2. 16782493

[91] Tang SY, Browne AJ, Mussell B, Smye VL, Rodney P. ‘Underclassism’ and access to healthcare in urban centres. Sociol Health Illn. 2015;37(5):698–714. 10.1111/1467-9566.12236. 25720520

[92] Thaddeus S, Maine D. Too far to walk: maternal mortality in context. Soc Sci Med. 1994;38(8):1091–110. 10.1016/0277-9536(94)90226-7. 8042057

[93] The A-M, Hak T, Koëter G, van der Wal G. Collusion in doctor-patient communication about imminent death: an ethnographic study. BMJ. 2000;321(7273):1376–81. 10.1136/bmj.321.7273.1376. 11099281 PMC27539

[94] Thomas JW, Penchansky R. Relating satisfaction with access to utilization of services. Med Care. 1984;22(6):553–68. 10.1097/00005650-198406000-00006. 6738145

[95] Tjepkema M, Bushnik T, Bougie E. Life expectancy of first Nations, Métis and inuit household populations in Canada. Health Rep. 2019;30(12):3–10. 31851367 10.25318/82-003-x201901200001-eng

[96] UNDP. Ten things to know about indigenous peoples. 2019. https://stories.undp.org/10-things-we-all-should-know-about-indigenous-people.

[97] United Nations. Resolution adopted by the general assembly on 20 December 2004 (A/RES/59/174). 2005. https://docs.un.org/en/A/RES/59/174.

[98] United Nations. United Nations declaration on the rights of indigenous peoples. 2007. https://www.converge.org.nz/pma/DRIPGA.pdf.

[99] United Nations. Indigenous peoples: health. 2019. https://www.un.org/development/desa/indigenouspeoples/mandated-areas1/health.html.

[100] Walker R, Pierre-Hansen N, Cromarty H, Kelly L, Minty B. Measuring cross-cultural patient safety: identifying barriers and developing performance indicators. Healthc Q. 2010;13(1):64–71. 10.12927/hcq.2013.21617. 20104040

[101] WHO. International agency for research on cancer: global cancer observatory statistics - Costa Rica. 2022. https://gco.iarc.who.int/media/globocan/factsheets/populations/188-costa-rica-fact-sheet.pdf.

[102] WHO. International agency for research on cancer: global cancer observatory statistics - Mexico. 2022. https://gco.iarc.who.int/media/globocan/factsheets/populations/484-mexico-fact-sheet.pdf.

[103] WHO. International agency for research on cancer: global cancer observatory statistics - Nicaragua. 2022. https://gco.iarc.who.int/media/globocan/factsheets/populations/558-nicaragua-fact-sheet.pdf.

[104] WHO. International agency for research on cancer: global cancer observatory statistics - world. 2022. https://gco.iarc.who.int/media/globocan/factsheets/populations/900-world-fact-sheet.pdf.

[105] WHO. NCDS progress monitor - Nicaragua. 2025. https://www.paho.org/sites/default/files/2020-03/NCDS-PROGRESS-MONITOR-2020-Nicaragua.pdf.

[106] WHO. Noncommunicable diseases - key facts. 2025. https://www.who.int/news-room/fact-sheets/detail/noncommunicable-diseases.

[107] Williams R. Cultural safety — what does it mean for our work practice? Aust N Z J Public Health. 1999;23(2):213–14. 10.1111/j.1467-842X.1999.tb01240.x. 10330743

[108] World Bank. Indigenous peoples. 2019. https://www.worldbank.org/en/topic/indigenouspeoples.

[109] World Bank. Indigenous peoples overview: poverty and health outcomes. 2025. https://www.worldbank.org/en/topic/indigenouspeoples.

[110] Zelčāne E, Pipere A. Finding a path in a methodological jungle: a qualitative research of resilience. Int J Qualitative Stud Health Well-Being. 2023;18(1):2164948. 10.1080/17482631.2023.2164948. PMC9828684 36606329

